# Towards a concept of disorders of “higher vestibular function”

**DOI:** 10.3389/fnint.2014.00047

**Published:** 2014-06-02

**Authors:** Thomas Brandt, Michael Strupp, Marianne Dieterich

**Affiliations:** ^1^German Center for Vertigo and Balance Disorders, University of Grosshadern MunichMunich, Germany; ^2^Clinical Neurosciences, University of Grosshadern MunichMunich, Germany; ^3^Department of Neurology, University of MunichMunich, Germany; ^4^Munich Cluster of Systems Neurology, SyNergyMunich, Germany

**Keywords:** central vestibular disorders, vestibular cognition, higher vestibular functions, room-tilt illusion, spatial neglect

## Abstract

**Background:** Vestibular disorders are commonly characterized by a combination of perceptual, ocular motor, postural, and vegetative manifestations, which cause the symptoms of vertigo, nystagmus, ataxia, and nausea. Multisensory convergence and numerous polysynaptic pathways link the bilaterally organized central vestibular network with limbic, hippocampal, cerebellar, and non-vestibular cortex structures to mediate “higher” cognitive functions.

**Anatomical classification of vestibular disorders:** The traditional classification of vestibular disorders is based on the anatomical site of the lesion. While it distinguishes between the peripheral and the central vestibular systems, certain weaknesses become apparent when applied clinically. There are two reasons for this: first, peripheral and central vestibular disorders cannot always be separated by the clinical syndrome; second, a third category, namely disorders of “higher vestibular function”, is missing. These disorders may be caused by peripheral as well as central vestibular lesions.

**Functional classification:** Here we discuss a new concept of disorders of higher vestibular function which involve cognition and more than one sensory modality. Three conditions are described that exemplify such higher disorders: room tilt illusion, spatial hemineglect, and bilateral vestibulopathy all of which present with deficits of orientation and spatial memory.

**Conclusions:** Further elaboration of such disorders of higher multisensory functions with respect to lesion site and symptomatology is desirable. The room tilt illusion and spatial hemineglect involve vestibular and visual function to the extent that both conditions can be classified as either disorders of higher vestibular or of higher visual functions. A possible way of separating these disorders in a first step is to determine whether the causative lesion site affects the vestibular or the visual system. For the vestibular system this lesion site may be peripheral or central. The criterion of “higher function” is fulfilled if cognition or senses other than the primarily affected one come into play.

## Introduction

The vestibular system is bilaterally organized: the otoliths act as sensors of gravity and linear head accelerations; the semicircular canals act as sensors of rotatory head accelerations. This input is distributed in a neuronal network that mediates perception of gravity and self-motion. The motor output of the vestibular system adjusts eyes, head, and body to an upright position in space. Vestibular pathways run from the labyrinth and the eighth nerve via the pontomedullary vestibular nuclei through ascending fibers to the ocular motor nuclei to mediate the vestibulo-ocular reflex (VOR). Once they reach the supranuclear eye-head coordination centers in the pontomesencephalic brainstem and the thalamus they are projected to several multisensory cortical areas in the temporo-parietal regions and the posterior insula for motion perception and spatial orientation. Animal studies have identified several distinct and separate areas of the temporo-parietal cortex that receive vestibular and somatosensory afferents, especially the core region of the parieto-insular vestibular cortex (PIVC; Grüsser et al., 1990a,b; Chen et al., 2011). Not only do these areas receive multisensory input, but they in turn directly project down to the vestibular nuclei (Akbarian et al., 1994). Thus, corticofugal feedback may modulate vestibular brainstem function. A homologue of the multisensory PIVC was found to be involved in middle cerebral artery infarctions, which cause deficits in the perception of verticality and self-motion (Brandt et al., 1994). Functional imaging with MRI and PET allows us to visualize a similar cortical vestibular network in humans which shows a dominance for vestibular cortical function in the non-dominant hemisphere when activated by caloric irrigation or galvanic stimulation of the peripheral vestibular system (Dieterich et al., 2003; Dieterich and Brandt, 2008) as well as by its functional connectivity (Kahane et al., 2003; zu Eulenburg et al., 2012).

Parallel input-output loops integrate the vestibulo-cerebellar structures. The vestibular system also modulates vegetative functions via ascending and descending pathways, e.g., from the vestibular nuclei to the locus coeruleus, and the central nucleus of the amygdala (Pompeiano et al., 2002) as well as the infralimbic cortex, and hypothalamus (Balaban and Thayer, 2001; Balaban, 2004). Further, numerous polysynaptic pathways link the vestibular nuclei with hippocampal and parahippocampal structures for spatial memory and navigation via the thalamus, the dorsal tegmental nucleus, or the pedunculopontine tegmental nucleus (Smith, 1997; Horii et al., 2004). Lesions along all these pathways–from the labyrinths to the cortex–may cause vestibular disorders. Their classification will be discussed below.

## Peripheral or central vestibular disorders?

**Figure 1:**
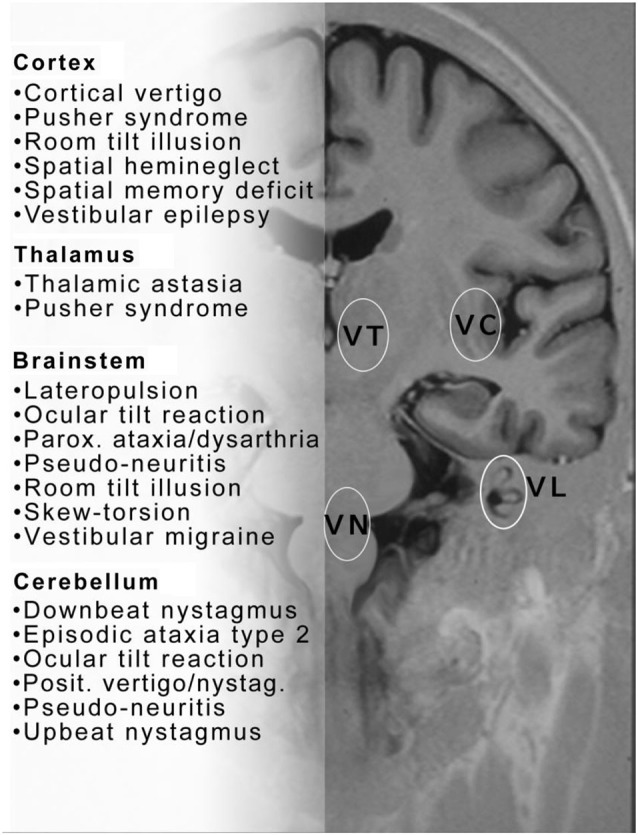
**This figure proposes a collection of clinical syndromes which may be called central vestibular disorders or disorders of higher vestibular function**. They are depicted in alphabetical order and topographically grouped for cerebral cortex, thalamus, brainstem and cerebellum. The topographic assignment remains uncertain for some conditions. Note also that similar disorders occur with lesions at different sites—brainstem or cortex (room tilt illusion) or brainstem and cerebellum ocular tilt reaction (OTR)—within the central vestibular neuronal circuitry. Please note that this list does not include all central vestibular syndromes. VC = vestibular cortex; VT = vestibular thalamus; VN = vestibular nucleus; VL = vestibular labyrinth.

The traditional classification of vestibular disorders is based on the anatomical site of the lesion and distinguishes between the peripheral vestibular system and the central vestibular system. The first includes the labyrinth and the vestibular nerve, i.e., the first- and second-order neurons. The latter involves central vestibular nuclei at the level of the pontomedullary brainstem and the pathways running from there to the vestibulo-cerebellum, brainstem, thalamus, and cortex areas (Figure 1). When applied clinically, this simple anatomical distinction suffers from certain weaknesses.

## A central lesion may mimick a peripheral disorder

The first weakness is the inaccuracy in diagnosing disorders originating at the transition from the peripheral to the central system, i.e., the root entry zone of the eighth nerve. This area is subserved by the second-order vestibular neurons traversing from the vestibular ganglion to the vestibular nucleus, and is “peripheral” by definition. Clinically, however, lesions of this fascicular region are caused by central pontomedullary brainstem disorders such as lacunar infarctions or multiple sclerosis (MS) plaques (Brandt, 1999; Kim and Lee, 2010). Thus, it is essential to differentiate peripheral vestibular neuritis from central vestibular “pseudoneuritis” already at the bedside in order to manage patients who present with signs and symptoms similar to those of acute prolonged vertigo. Whereas acute vestibular vertigo with spontaneous nystagmus and a pathological head-impulse test are typical for an acute peripheral failure, a normal head-impulse test, especially when combined with skew deviation of the eyes, indicates a central origin (Cnyrim et al., 2008; Newman-Toker et al., 2008; Kattah et al., 2009; Kim and Lee, 2012). However, it is important to note that a pathological head-impulse test can also be found in central lesions affecting the vestibular nuclei and even the cerebellum, thus mimicking a peripheral vestibular lesion (Cnyrim et al., 2008).

## Different lesion sites along vestibular pathways can cause the same syndrome

Peripheral disorders cause vestibular syndromes that are commonly characterized by a combination of perceptual, ocular motor, postural, and vegetative manifestations: vertigo, nystagmus, ataxia, and nausea (Brandt, 1999). Patients with central disorders may present with only single components like tilts of the perceived vertical or lateropulsion without vertigo and nystagmus. This arises if the lesion site is within the network of nuclei and pathways, which may cause ocular motor disorders in brainstem lesions or perceptual disorientation in cortical lesions.

**Figure 2:**
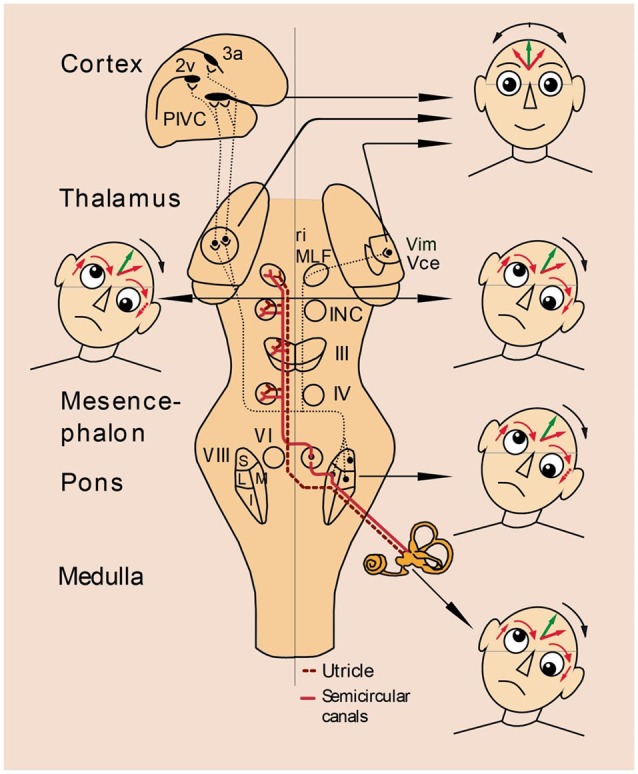
**Vestibular syndromes in the roll plane: graviceptive pathways from the otoliths and the vertical semicircular canals mediating vestibular function in the roll plane**. The projections from the otoliths and the vertical semicircular canals to the ocular motor nuclei (trochlear nucleus IV, oculomotor nucleus III, abducens nucleus VI), the supranuclear centers of the interstitial nucleus of Cajal (INC), and the rostral interstitial nucleus of the MLF (riMLF) are shown. They subserve the VOR in three planes. The VOR is part of a more complex vestibular reaction, which also involves vestibulospinal connections via the medial and lateral vestibulospinal tracts for head and body posture control. Furthermore, connections to the assumed vestibular cortex (areas 2v and 3a and parieto-insular vestibular cortex, PIVC) via the vestibular nuclei and the thalamus (Vim, Vce) are depicted. Graviceptive vestibular pathways for the roll plane cross at the pontine level. The ocular tilt reaction (OTR; with skew torsion, head tilt, and tilt of perceived vertical) is depicted schematically in relation to the level of the lesion: ipsiversive OTR with peripheral and pontomedullary lesions (bottom two of the heads on the right); contraversive OTR with pontomesencephalic lesions (head on the left). In cases of lesions of the vestibulothalamic tract isolated tilts of SVV may be ipsiversive and in vestibular thalamic lesions, the tilts of SVV may be contraversive or ipsiversive; in vestibular cortex lesions, they are preferably contraversive (top two of the heads on the right). OTR is not induced by supratentorial lesions above the level of INC.

Another weakness of a purely anatomical distinction between peripheral and central vestibular disorders involves the sensitivity and specificity of the attribution of a specific dysfunction to a lesioned structure. Such attribution is easier to do for peripheral than for central disorders. For example, a “peripheral” canalolithiasis and cupulolithiasis of the horizontal or vertical semicircular canals can be unambiguously determined by the direction of the positioning nystagmus. In contrast, a “central” vertical divergence of the visual axes of the eyes (skew deviation) is ambiguous because it may originate in various lesion sites. Skew deviation occurs with unilateral lesions of vestibular pathways at different levels–from the vestibular nuclei to the midbrain tegmentum and the vestibulo-cerebellum (Brandt and Dieterich, 1994). Management of the thus afflicted patients, however, requires precise topographic diagnostic tools in order to identify the structures affected and the lesion’s extent. They determine not only the actual neurological deficit but also allow prediction of recovery and long-term outcome. Thus, for a topographic diagnosis of vestibular brainstem and cerebellar syndromes it is necessary to seek additional neurological signs and symptoms. For example, due to the pontomedullary crossing of ascending vestibular pathways, it is helpful to identify the direction of skew deviation or the tilt of the subjective visual vertical (SVV; Figure 2); they indicate the side of the lesion, if the level of brainstem disorders is known or the level of the lesion, if the affected side is known (Dieterich and Brandt, 1993; Brandt and Dieterich, 1994; Zwergal et al., 2008; Baier et al., 2012).

In brief, despite some weaknesses it is logical, on the one hand, to classify peripheral and central vestibular dysfunctions according to anatomy. On the other, an additional category based on cognitive signs and symptoms is required.

## Higher vestibular functions

There are additional “higher” aspects of central vestibular function and dysfunction which result from the integration of the vestibular network in cognitive functions at the cortical level and within the hippocampal and limbic system. These aspects comprise the internal representation of the body schema and the internal model of the surrounding space as well as multisensory motion perception, attention, spatial memory, and navigation. These functions can be termed “higher vestibular functions” in analogy with the well-established term “higher visual functions” in neuroophthalmology. The latter term correlates circumscribed cortical lesions with particular dysfunctions of “higher visual perception”, such as recognition, memory, or spatial orientation with lesions along the ventral and dorsal streams of visual input. These streams are also called the “what” and “where” pathways (de Haan and Cowey, 2011). They seem to reflect a division of labor that is made between vision-for-action by the dorsal stream and vision-for-perception by the ventral stream (Milner and Goodale, 2008; Goodale, 2011). Accordingly, disorders of higher vestibular function are characterized by complex perceptual, sensorimotor, and behavioral deficits that exceed basic perceptions of head acceleration or motor responses, such as the vestibulo-ocular or vestibulo-spinal reflexes. The cognitive neurology of the vestibular system is being increasingly acknowledged nowadays (Seemungal, 2014). However, a description of disorders of “higher vestibular function” has not yet been elaborated, although there is experimental evidence. For example, the pusher syndrome and “visuo-spatial” hemineglect—which are not primarily considered vestibular disorders–are in some aspects related to vestibular function (Brandt, 1999; Karnath and Dieterich, 2006).

In the following we will discuss three conditions as potential candidates that will help to elaborate a classification of higher vestibular disorders, namely the room tilt illusion, spatial hemineglect, and impairment of spatial memory and navigation in bilateral vestibular loss. These three conditions will help us to elucidate the unique features of higher vestibular disorders in contrast to those of higher disorders of other senses like vision or hearing. In the process the difficulties and limitations of such attempts to nosologically separate these disorders from other sensory modalities and even from peripheral vestibular disorders will also become overt.

## Disorders of higher vestibular function versus disorders of higher visual function

There are similarities and differences between the higher sensory disorders of visual and vestibular function. They are similar in that both manifest with cognitive disturbances of spatial orientation, attention, spatial memory, and navigation. They are typically different in the following ways:

- In higher visual disorders the lesion site is mostly within the visual cortex, and the symptomatology arises from a dysfunction of the visual cortex. However, the hierarchically organized cortical visual network is much more complex and larger than that of the vestibular cortex.
- In higher vestibular disorders the lesion site may involve the vestibular cortex, but it may also be located in the subcortical vestibular circuitry or even in the peripheral end-organs.
- Higher vestibular disorders often involve dysfunction of other sensory modalities so that the same disorder can be called a higher vestibular, higher visual, or higher somatosensory disorder.
- Some higher vestibular dysfunctions are only the cognitive symptoms caused by a peripheral vestibular disorder.

All of the above listed features are best illustrated by the rare vestibular syndrome of room tilt illusion.

## Room tilt illusion

**Figure 3:**
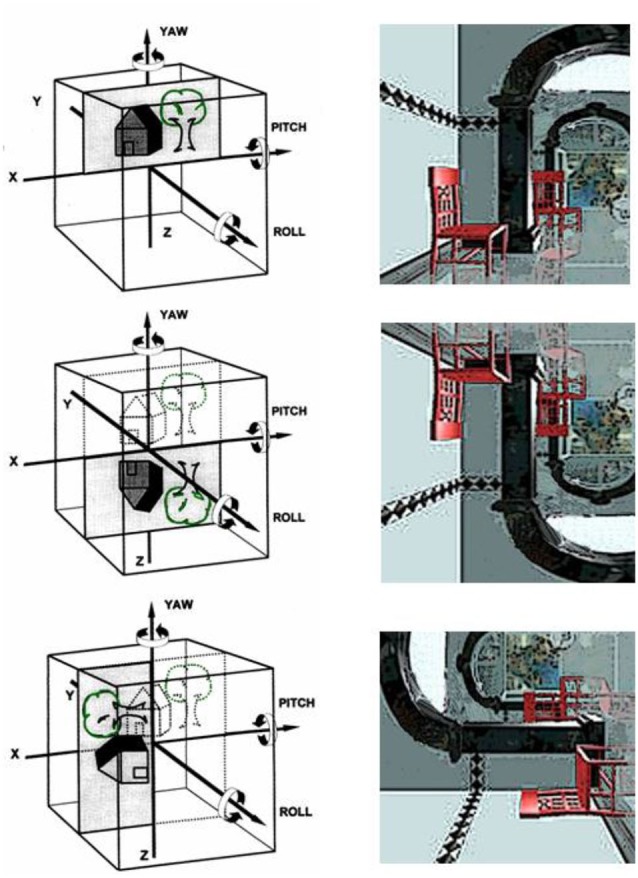
**Room-tilt illusion: schematic representation of the head as a cube with the cortical matching of the vestibular and the visual 3-D coordinate maps**. The three major planes of action of the vestibular system are the frontal roll, the horizontal yaw, and the sagittal pitch about the *x, y* and *z* axes, respectively. (Top) Visual scene matches with the vestibular coordinates; (middle) room tilt illusion with 180^°^ tilted visual scene in the pitch plane (upside-down vision); (bottom): room tilt illusion with 90^°^ tilt in the roll plane (modified from Brandt, 1997).

Transient upside-down inversion of vision—the room tilt illusion—has been repeatedly described in patients with lower brainstem infarctions (Ropper, 1983; Tiliket et al., 1996; Sierra-Hidalgo et al., 2012) or with cortical lesions (Solms et al., 1988), especially in cases of vestibular epilepsy (Smith, 1960). These illusions last for seconds or minutes, rarely up to hours. They are often associated at the beginning with rotational vertigo, and recovery is either rapid or involves a gradual uprighting to normal position. Transient upside-down vision or 90° tilts are obviously vestibular signs that indicate a misperception of verticality. Spatial orientation of verticality is based on the interactions between the visual and vestibular systems. Both senses provide us with cues about vertical orientation in 3-D coordinates. The visual and vestibular cortices have to match vestibular spatial coordinates in three dimensions with the orientation of the visual scene to determine the unique egocentric perception of right and left, up and down, and fore and aft. It is not possible to perceive two different verticals, a visual and a vestibular one, at the same time. In brief, room-tilt illusions are, in our opinion, transient mismatches of the visual and vestibular 3-D map coordinates that occur in 90° or 180° steps (Brandt, 1997). They are the erroneous result of an attempted cortical match (Figure 3).

This condition corresponds with several of the above-described typical features that distinguish higher vestibular from higher visual disorders. First, the lesion site is mostly subcortical, i.e., within the brainstem or the peripheral end-organ (e.g., Meniere’s disease, bilateral vestibular failure). Second, the cause of the disease is vestibular; the symptomatology, however, is visual. Third, the clinical syndrome of room tilt illusion could be classified as either a higher vestibular or a higher visual disorder.

## Spatial neglect

We hypothesize that the mechanisms of visuo-spatial neglect are predominantly elicited by a vestibular tonus imbalance (Brandt, 1999; Karnath and Dieterich, 2006; Brandt et al., 2012). Spatial neglect is a disorder of spatial attention and orientation; awareness of visual stimuli is disrupted and occurs in one egocentric hemifield that is contralateral to an acute temporo-parietal lesion of the (most often) right hemisphere (Vallar and Perani, 1986). Patients so afflicted may have preserved visual fields, but they spontaneously direct their spatial attention and eye and head movements to the ipsilesional hemifield. This results in a visuospatial neglect of stimuli in the contralateral hemifield. Karnath and Rorden (2012) stress the “heterogeneous collection of symptoms with controversial anatomical correlates”. They also draw attention to biased gaze deviation and search, mainly due to lesions of the right hemisphere perisylvian region, and object-centered deficits (line bisection), caused primarily by more posterior and inferior lesions.

**Figure 4:**
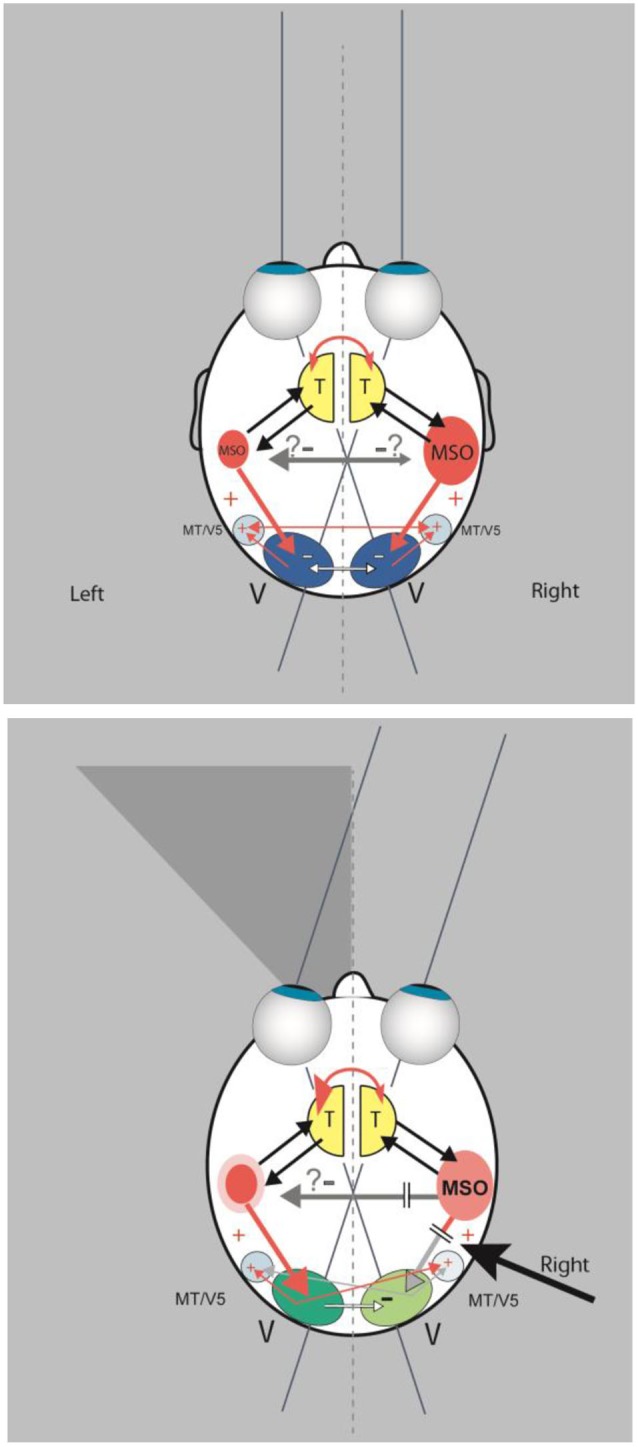
**(Top) In this scheme a double organization of the spatial attention and orientation center, represented by an “MSO” (multisensory orientation) in each hemisphere, is assumed, as is a dominance of the right hemisphere**. Interhemispheric transcallosal connections are inhibitory. The MSO receives vestibular and somatosensory input from the thalamus (T) and directs visual attention by excitatory connections to the ipsilateral or contralateral visual cortex (V). The schematic drawing shows also transcallosal connections between the visual cortices. They are mainly inhibitory (white arrows) and to a lesser extent excitatory (thin red arrows) for bilateral activation of the motion-sensitive areas MT/V5. **(Bottom)** A lesion of the dominant MSO in the right hemisphere causes a left-sided visuo-spatial neglect due to less excitation (“deactivation”) of the ipsilateral visual cortex. This is further suppressed by increased inhibition from the contralateral visual cortex. While the motion-sensitive area MT/V5 receives less input from the ipsilateral visual cortex, it still receives excitatory input from the contralesional MT/V5. Modulation of the left visual cortex could hypothetically result in enhanced visuo-spatial attention within the right hemifield due to the increased activation from the nondominant MSO and less inhibition from the ipsilateral right visual cortex (modified from Brandt et al., 2012).

Imaging techniques in patients with neglect provided evidence that the cortical areas involved are the superior temporal cortex, the insula, the temporo-parietal junction (Karnath and Dieterich, 2006), and the middle frontal gyrus and the posterior intraparietal sulcus (Ptak and Schnider, 2011). Some of these structures are core regions of the cortical multisensory vestibular network (Brandt and Dieterich, 1999; zu Eulenburg et al., 2012). It has been found that the dominance for vestibular cortical function lies in the nondominant hemisphere, i.e., the right hemisphere in right-handers (Dieterich et al., 2003). Studies showing that vestibular (caloric) stimulation significantly improved spatial functioning have demonstrated the important role of the vestibular system in neglect (Cappa et al., 1987; Vallar et al., 1993). For example, when vestibular stimulation was combined with neck muscle vibration, the horizontal deviation combined linearly, adding or neutralizing the effects observed during application of both types of stimulation (Karnath, 1994). Therefore, the question arose as to whether spatial neglect is a disorder of the “multisensory vestibular cortex” (Brandt, 1999; Karnath and Dieterich, 2006). In Figure 4 a schematic drawing depicts the major anatomical structures involved and their functional connections as the basis for a hypothetical model of certain underlying mechanisms (Brandt et al., 2012).

This condition is also in line with several of the above-described features. First, the lesion site is in the vestibular cortex of the right hemisphere, which is the dominant hemisphere for the vestibular system in right-handers. However, neighboring non-vestibular structures of the temporo-parietal cortex and the thalamus are also involved, i.e., a lesion restricted to the vestibular cortex does not cause hemineglect. Second, the symptomatology involves visual and somatosensory perception as well as ocular motor exploration and eye-hand coordination. Third, the clinical syndrome could be equally classified as a higher vestibular, a higher visual, or a higher somatosensory disorder.

## Bilateral vestibular loss with spatial memory deficit

Key symptoms of bilateral vestibulopathy are (i) movement-dependent postural dizziness and unsteadiness of gait and stance (exacerbated in the dark and on unlevel ground); they are absent when sitting or lying; (ii) blurred vision when walking and during head movements (oscillopsia); and (iii) impaired spatial memory and navigation (Brandt et al., 2013). Patients mostly complain about postural vertigo and gait unsteadiness when moving. They are typically free of symptoms under static conditions, i.e., when sitting or lying. About 40% of those affected notice illusory movements of the surroundings (oscillopsia) while walking or running, and consequently can no longer read street signs or definitely identify the faces of people approaching them.

An intact vestibular function is important for spatial orientation, spatial memory, and navigation (Smith, 1997). Patients with bilateral vestibulopathy have significant deficits of spatial memory and navigation (tested with a virtual variant of the Morris water task) as well as atrophy of the hippocampus (Brandt et al., 2005), but the rest of their memory functions are not affected. The latter was tested by the Wechsler Memory Scale-Revised in full which constitutes the most universally employed memory test battery (Brandt et al., 2005). Patients with unilateral labyrinthine failure, however, do not have significant disorders of spatial memory or atrophy of the hippocampus (Hüfner et al., 2007). Spatial navigation requires a continuous representation of the location and motion of the individual within a 3-D environment, whose coordinates are provided mainly by vestibular and visual cues. Consequently hippocampal atrophy may impair complex forms of spatial memory processing, while non-spatial functions remain well preserved. Perhaps the ancient phylogenetic role of the hippocampus in spatial memory processing (Kessels et al., 2004), which requires an intact vestibular input, is more sensitive to hippocampal atrophy than more advanced, non-spatial roles that rely additionally on the surrounding medial-temporal lobe and prefrontal tissue (Markowitsch et al., 2003).

This disorder especially reflects the last of the four features that distinguish higher vestibular from higher visual disorders. Bilateral vestibular loss is a well-defined peripheral disorder of both labyrinths or vestibular nerves. Impaired spatial memory, orientation, and navigation are additional higher vestibular symptoms, i.e., cognitive consequences of the absent vestibular input.

## Conclusions

The three above-described syndromes represent cognitive disorders of higher vestibular function, a clinically desired third category of vestibular disorders in addition to the traditional distinction between peripheral and central vestibular disorders. They involve not only convergence of multisensory input but also of sensorimotor integration with spatial memory, orientation, attention, navigation, and the interaction of body and surround during locomotion. Other examples are vestibular epilepsy, the pusher syndrome, thalamic astasia, or lateropulsion with tilts of perceived verticality. Some may manifest as paroxysms or transient episodes such as vestibular epilepsy and room tilt illusion. Some resolve spontaneously or within days to weeks with the support of physical therapy, as in pushing behavior. Sometimes patients recover but have residual deficits such as extinction in visuospatial hemineglect. The causative lesion is not necessarily restricted to cortical structures; an example of this is the room tilt illusion, which may be elicited by peripheral or central vestibular dysfunctions originating from the labyrinth to the vestibular cortex. Disorders of higher vestibular function can manifest as a consequence of a peripheral vestibular failure, e.g., deficits in orientation, spatial memory, and navigation in bilateral vestibular loss.

An elaboration of a classification of disorders of higher vestibular function has to consider multisensory convergence, which for the vestibular system—in contrast to the visual or auditory systems–already occurs at the level of the vestibular nuclei. The vestibular cortex is not a “primary sensory cortex” like the visual cortex. All vestibular cortex neurons are multisensory and respond to stimuli of various modalities. Disorders of higher vestibular or higher visual function could be separated by the lesion site by determining whether it affects the vestibular or the visual system. Such a distinction is, however, clinically unsatisfying, especially when the symptomatology is dominated by a dysfunction of another sensory modality. This is the case for the room tilt illusion in which the lesion site is in the vestibular system, but the symptomatology of an upside-down inversion of vision is in the visual system. Thus, some conditions can be classified as either higher vestibular or higher visual dysfunction depending on the classifying criterion, which can be the site of the lesion or the symptomatology. A comprehensive elaboration of disorders of all higher sensory functions is still necessary.

## Conflict of interest statement

The authors declare that the research was conducted in the absence of any commercial or financial relationships that could be construed as a potential conflict of interest.
